# Avoiding off-target effects in electrical stimulation of the cervical vagus nerve: Neuroanatomical tracing techniques to study fascicular anatomy of the vagus nerve

**DOI:** 10.1016/j.jneumeth.2019.108325

**Published:** 2019-09-01

**Authors:** Nicole Thompson, Svetlana Mastitskaya, David Holder

**Affiliations:** Department of Medical Physics and Biomedical Engineering, University College London, London, United Kingdom

**Keywords:** Vagus nerve, Neuronal tracers, Electroceuticals, Vagus nerve stimulation, Off-target effects, Neuroanatomical mapping, Neuroanatomy

## Abstract

Vagus nerve stimulation (VNS) is a promising therapy for treatment of various conditions that are resistant to standard medication, such as heart failure, epilepsy, and depression. The vagus nerve is a complex nerve providing afferent and efferent innervation of the pharynx, larynx, heart, tracheobronchial tree and lungs, oesophagus, stomach, liver, pancreas, small intestine and proximal colon. It is therefore a prime target for intervention for VNS. Surprisingly, the fascicular organisation of the vagus nerve at the cervical level is still not well understood. This, along with the current stimulation techniques, results in the entire nerve being stimulated, which leads to unwanted off-target effects. Neuronal tracing is a promising method to delineate the organ-specific innervation by the vagus nerve, thereby providing valuable insight into the fascicular anatomy. In this review we discuss the current knowledge of vagus nerve anatomy and neuronal tracers used for mapping of its organ-specific projections in various species. Efferent vagal projections are a chain of two neurones (pre- and postganglionic), while afferent projections consist of only one pseudounipolar neurone with one branch terminating in the target organ/tissue directly and another in the brainstem. It would be feasible to retrogradely trace the afferent fibres from their respective visceral targets and identify them at the cervical level using non-transsynaptic neuronal tracers. Using this to create a map of the functional anatomical organisation of the vagus nerve will enable selective VNS ultimately allowing for the avoidance of the off-target effects and improving overall efficacy.

## Highlights

• The vagus nerve provides innervation to the majority of the visceral organs.
• There is a need to delineate fascicular anatomy to avoid off-target effects of VNS.
• Neuronal tracing is a powerful tool to study fascicular organization.

## 1 Introduction – Neuronal tracing for delineation of vagal fascicular anatomy

Pharmacological therapy and surgical intervention are the most commonly used approaches for treatment of various pathologies (Mishra, 2017). However, definite side effects continue to prevail for all known drugs, surgeries and non-surgical interventions (de Boer et al., 2013; Dunlop, 1969; Zimmerman, 1999). The brain, nervous system and endocrine system (via neural impulse communications through complex neural circuitry) regulate functions of all internal organs. Most drugs act on or interfere with neurotransmitters and their receptors, or endocrine system mechanisms (Locatelli et al., 2009). In order to avoid these side effects, the new field of bioelectronics medicine, Electroceuticals, was born. Electroceuticals employs electrical stimulation of nerves to treat diseases instead of administering drugs or performing complex surgical procedures (Famm et al., 2013; Kollewe, 2017; Mishra, 2017). Electrical impulses (or action potentials) are the language of the nervous system which result in regulation of virtually all organs and functions (Famm et al., 2013; “The Brain and Nervous System,” 2006). Electroceuticals can modulate these neural impulses to recover lost function and revive a healthy balance (Famm et al., 2013; Mishra, 2017).

A prime target for intervention is the cervical vagus nerve (Blount, 2015; Ekmekçi and Kaptan, 2017; Guiraud et al., 2016; Koopman et al., 2016; Pečlin and Rozman, 2014; Smucny et al., 2015). The vagus nerve, also known as cranial nerve X, innervates numerous visceral organs and muscles; including the pharynx, larynx, heart, lungs, muscles of the bronchi and gut. The human cervical left and right vagi consist of approximately 10–15 individual fascicles (Verlinden et al., 2016), but, surprisingly, the organisation of fascicles, functional, anatomical or other, within the vagus nerve remains almost completely unknown. The vagus nerve follows a complex anatomical path and crosses several plexuses and ganglia along its thoracoabdominal course. Electrical stimulation of the cervical vagus nerve has been successfully used to reduce depression, arthritis and the frequency of epileptic seizures (Binnie, 2000; De Ferrari and Schwartz, 2011; Ripplinger, 2017). At the moment, however, stimulation techniques and lack of understanding of fascicular anatomy allow for only the entire nerve to be activated or suppressed. Therefore, with the considerable innervation by the vagus nerve, whole nerve stimulation inevitably leads to off-target side effects with organs other than those intended being stimulated (Ripplinger, 2017). Knowledge of the functional anatomy of fibres could be helpful not only in avoiding side effects but also in improving the efficacy of vagus nerve stimulation (VNS) overall by better understanding of the mapping of vagal fibres to both peripheral organs and originating brain regions.

In our research group, we are developing methods to enable selective stimulation of fascicles within the cervical vagus nerve (Aristovich et al., 2019). We are currently investigating the functional anatomy of the vagus nerve using neural tracers, electrophysiology with multielectrode arrays, computerised tracing with microCT and the new method of fast neural Electrical Impedance Tomography (EIT). This review has arisen from this work as we strive to understand the fascicular functional anatomy of the vagus nerve in order to allow selective VNS and avoid off-target effects.

With a known fascicular organisation of the vagus nerve, selective stimulation could be used to target specific organs or effectors for neuromodulation and avoid adverse off-target effects. Preliminary studies have shown this is possible using cuff electrodes optimised for selective stimulation in sheep, dogs and humans (Aristovich et al., 2019; Pečlin et al., 2009; Rozman and Bunc, 2004). Stimulation through a pair of electrodes in the same radial position on two rings of 14 electrodes in a cylindrical multielectrode nerve cuff array in the sheep allows for selective neuromodulation of cardiac, pulmonary and recurrent laryngeal activity in the vagus nerve; evidence is yet to be obtained to show that function is localised to individual fascicles, but these results already suggest that such function is localised within the nerve (Aristovich et al., 2019). Corresponding localised activity could be imaged using the same nerve cuff and fast neural EIT (Aristovich et al., 2019). It is thus hypothesized that there is functional organotopic organisation of the fascicles in the cervical vagus nerve (Aristovich et al., 2019; Prechtl and Powley, 1987); the evidence for which is the three notable regions identified in the above mentioned research: cardiac, pulmonary and recurrent laryngeal. To account for inter-individual differences, tracing techniques in animal studies may enable estimation of the degree of variation; however, the non-invasive EIT technique could be used to provide subject-specific fascicular mapping and so inform targeted stimulation.

Neuroanatomical tracing with neuronal markers is a widely used technique for tracing projections in the central nervous system (CNS) and, to a lesser extent, has also been applied in the peripheral nervous system (PNS). In theory, it could be helpful in delineating the functional anatomy in the human autonomic nervous system (ANS). However, it requires continuity of the neuronal processes and may become ineffective for tracing axons over the long distances needed for autonomic nerves in large mammals such as the human. The question remains whether this would be feasible for tracing fascicles in the cervical vagus nerve, considering the complexity of the vagus nerve anatomical path. In this review, we discuss the current knowledge of functional anatomical organisation of the vagus nerve and plausible techniques for neuroanatomical tracing of its individual organ-specific fascicles to ascertain the fascicular organisation.

## 2 Purpose

Neuroanatomical mapping of organ-specific projections of the vagus nerve would require imaging and tracing of the afferent and efferent fascicles. First, we review and discuss studies of vagal innervation in humans and experimental animals. Next, we discuss the strategies to choose the appropriate neuronal tracer(s) based on their mechanisms of action, axonal transport and the targeted projections (afferent or efferent). We conclude with recommendations on the most appropriate tracing approaches.

## 3 Vagus nerve

**Fig. 1:**
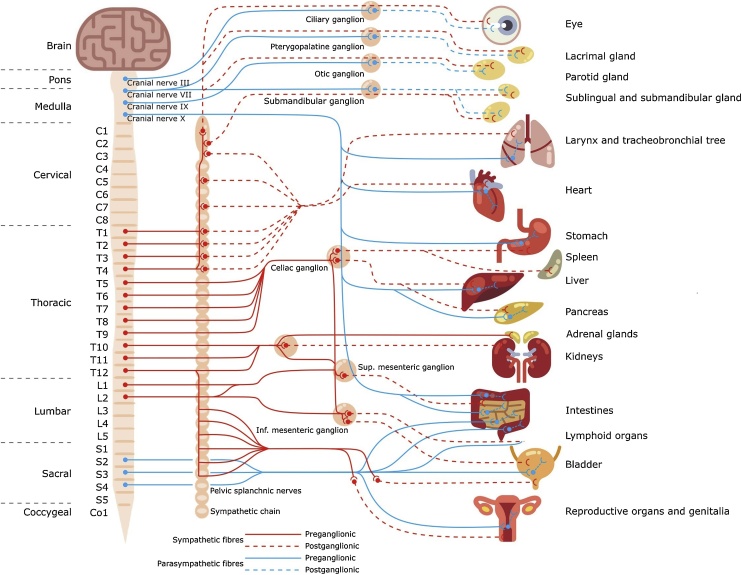
General schema of the autonomic nervous system.

The ANS, a division of the PNS, comprises sympathetic and parasympathetic divisions (Fig. 1) (Felten et al., 2016; Waxman, 2017). Often described as the visceral system, the ANS comprises visceral afferent (sensory) and visceral efferent fibres; the latter are either parasympathetic or sympathetic (Amann and Constantinescu, 1990). The visceral efferent division is a two-neurone chain with preganglionic neurones which arise from the brainstem or spinal cord and synapse on postganglionic neurones in various types of ganglia which connect them to visceral target organs (Felten et al., 2016; Marieb, 2010). The sympathetic nervous system is a thoracolumbar system derived from neurones in the T1-L2 lateral horn. It prepares the body for fight-or-flight reactions by acting through sympathetic chain and collateral ganglia (e.g. coeliac ganglia) (Marieb, 2010; McCorry, 2007). The parasympathetic division, also known as the craniosacral system, consists of cranial nerves (CNs III, VII, IX and X) and spinal nerves in the sacrum (S2-S4) (Felten et al., 2016). All CNs arise from brainstem and CNS nuclei, and CNs III, VII and IX synapse at cranial nerve ganglia (ciliary, pterygopalatine, otic or submandibular) before completing their journey to target tissues via trigeminal branches (Türkmen et al., 2015; Watson, 2012). Unlike the other cranial nerves, the vagus nerve (CN X), along with the sacral nerves that arise from intermediate grey matter in the spinal cord, acts through intramural ganglia which lie within or near the target tissue (Rea, 2014; Sibilla and Agarwal, 2018). The parasympathetic nervous system is a regulatory and homeostatic reparative system responsible for rest-and-digest activity stimulation when the body is at rest (McCorry, 2007). Both autonomic divisions work together with most connections acting through vagal and sympathetic preganglionic neurones (Felten et al., 2016).

The vagus nerve is the main nerve of the parasympathetic division of the ANS (Pavlov and Tracey, 2012). However, it is a mixed nerve containing branchial motor, visceral sensory, visceral motor, and special and general sensory components (Rea, 2014). The vagus nerve contains 80 to 90% afferent fibres (sensory) and 10 to 20% efferent (motor) fibres (Beckett et al., 2017). It is the longest cranial nerve with extensive distribution below the level of the head (Felten et al., 2016; Rea, 2014; Sibilla and Agarwal, 2018). Despite the gross anatomy of the vagus nerve being well studied, its fascicular functional anatomy remains almost completely unknown. The vagus nerve includes axons which emerge from or converge onto four nuclei of the brainstem: the **dorsal nucleus of the vagus nerve** (DVMN) – which sends parasympathetic motor output to intramural ganglia associated with thoracic and abdominal viscera and supplies autonomic innervation to the heart, the lungs, and the gastrointestinal tract (GIT) to the descending colon, the **nucleus ambiguus** – which gives rise to the branchial efferent motor fibres of the vagus nerve and preganglionic parasympathetic neurones that innervate the heart, the **solitary nucleus** (NTS) – which receives afferent taste information and primary afferents from visceral organs, and the **spinal trigeminal nucleus** – which receives information about deep/crude touch, pain, and temperature of the outer ear, the dura of the posterior cranial fossa and the mucosa of the larynx (Câmara and Griessenauer, 2015; Felten et al., 2016; Rea, 2014).

In addition, vagal brainstem nuclei establish multiple central afferent and efferent connections in brain regions involved in affective autonomic and other interoceptive reflexive functions. Major central afferent connections from the NTS are derived from hypothalamus and central nucleus of amygdala. In turn, the NTS sends the axonal projections terminating in numerous autonomic brain regions, such as brainstem reticular formation, parabrachial nucleus, periaqueductal grey, amygdala, and hypothalamus. Therefore, the NTS provides the autonomic affective functional basis for homeostatic control of the whole body (Nieuwenhuys et al., 2008). The nucleus ambiguus and DVMN also receive multiple projections from various brain regions, including Raphe nuclei and hypothalamus (Manaker and Fogarty, 1995; Roges et al., 1980), thus providing an anatomic substrate for central modulation of parasympathetic function.

From the origin in the brainstem in the CNS (Hermanowicz, 2007), the filaments of the vagus nerve travel towards the jugular foramen where the vagus nerve runs through caudal to the glossopharyngeal nerve and superficial to the internal jugular vein (Ellis, 2006; Waldman, 2011). Within and inferior to the jugular foramen lie the two ganglia associated with the vagus nerve, the **superior (jugular)** and **inferior (nodose) ganglia**, respectively (Câmara and Griessenauer, 2015; Ellis, 2006; Rea, 2014). Both motor and sensory fibres pass through the jugular ganglion, whilst only some sensory fibres (namely, visceral afferent) have cell bodies in the nodose ganglion. The jugular ganglion contains the cell bodies of the general somatic afferent fibres, specifically from the external ear (Rea, 2014). The nodose ganglion is primarily concerned with visceral afferent fibres and their cell bodies for the relaying of information from the pharynx and thoracic and abdominal viscera (Câmara and Griessenauer, 2015; Rea, 2014). The left and right vagus nerves subsequently descend in their own anterolateral compartments, the carotid sheaths, and run between the internal jugular vein and the internal and external carotid arteries, and continue into the thorax and abdomen where they innervate the heart, tracheobronchial tree and lungs, oesophagus, stomach, pancreas, liver and GIT via numerous branches.

There are also sympathetic ganglia which are anatomically close to vagal nerve branches. These are the collateral ganglia of the sympathetic nervous system which include the coeliac ganglion and the superior and inferior mesenteric ganglia. The collateral ganglia lie close to the vagal nerve path and are often confused to be vagus nerve ganglia. Parasympathetic preganglionic neurones may appear to pass through the collateral ganglia, especially when traveling through a plexus; however, no synapses take place. Therefore, they are anatomically and functionally separate. Sympathetic preganglionic axons, as part of the spinal nerves, leave the CNS, pass through the sympathetic chain ganglia and synapse in these collateral or prevertebral ganglia; whilst some synapse already with postganglionic neurones within the sympathetic chain ganglia and others pass through all ganglia and continue to the adrenal glands (Furness, 2015; Gibbins, 2012; Szurszewski and Linden, 2012; Waldman, 2009).

The cell bodies of the efferent parasympathetic preganglionic neurones are located in the abovementioned DVMN and nucleus ambiguus of the brainstem (Watson, 2012; Weaver, 1980). The cell bodies of parasympathetic postganglionic neurones are located within the intramural ganglia (Furness, 2009; Johnson, 2013) – ganglia so named for their positioning within or near the organs they innervate (Langley, 1921). Efferent fibres pass through the jugular and nodose ganglia without synapsing. Therefore, there are synapses between the pre- and postganglionic visceral efferent fibres occurring only within the intramural ganglia near to the target organ (McCorry, 2007; Molnar and Gair, 2013).

Visceral afferent fibres are pseudounipolar with the neurone containing an axon that splits into two branches; from the cell bodies, which are collectively located in the jugular and nodose ganglia as mentioned above, the peripheral branches extend uninterrupted to the periphery and the central ones to the CNS where they centrally synapse in the solitary and spinal trigeminal nuclei (Varga and Mravec, 2015). Therefore, there are no synapses of the visceral afferent fibres other than in the target organ or in the nuclei of the brainstem of the CNS.

Various types of neurones form signalling circuits such as a reflex arc. Multiple afferent and efferent neurones are connected by interneurones which are exclusively located within the CNS. This allows for one motor neurone to be affected by multiple sensory neurones and one sensory neurone to affect multiple motor neurones (Lodish et al., 2000). When a reflex involves only one central synapse within the CNS between afferent and efferent neurones, this is known as monosynaptic. The vagus nerve makes use of polysynaptic reflexes which involve one or more interneurones (Craighead and Nemeroff, 2000).

In summary, the vagus nerve sends **efferent parasympathetic activity** via the preganglionic neurones originating in the DVMN and the nucleus ambiguus that project to the terminal intramural ganglia of the thoracic and abdominal cavities which lie within or near to the target organs. Here, they synapse with postganglionic neurones that project from the intramural ganglia a short distance to the target effector, or to the specific target tissue within the organ. The vagus nerve then **conveys information from visceral tissues and organs via afferent sensory fibres** projecting directly from the target organs to the solitary and spinal trigeminal nuclei of the brainstem, with the cell bodies of these neurones located in the inferior and superior cervical ganglia of the vagus nerve. Interneurones located within the CNS relay the information from the afferent fibres to the efferent fibres allowing integration of sensory information at the level of brainstem and control of parasympathetic efferent output to periphery to maintain body homeostasis.

### 3.1 Innervation and functions of the vagus nerve

The vagus nerve has efferent and afferent functions. With regards to the former, the vagus nerve innervates, through a motor component, the majority of the muscles associated with the pharynx and larynx via the pharyngeal branch and the superior and recurrent laryngeal nerves, respectively (Felten et al., 2016; Rea, 2014). Additionally, a large part of the efferent function is the parasympathetic supply to the visceral organs. Those well-known to receive parasympathetic innervation from the vagus nerve include: the heart, tracheobronchial tree and lungs, oesophagus, stomach, liver, pancreas, small intestine and proximal colon (Agostoni et al., 1957; Câmara and Griessenauer, 2015; Hermanowicz, 2007; Pavlov and Tracey, 2012; Rea, 2014; Sibilla and Agarwal, 2018; Waldman, 2011). Thoracic and abdominal communicating branches between the left and right vagal trunks have been described in some species, with afferent and/or efferent axons from one side crossing over to the other side. In the rat, there is little evidence for crossing of efferent axons, while about 20% of afferents cross from one side to another at the thoracic level (Berthoud and Neuhuber, 2000).

The vagal innervation of kidneys, spleen, adrenal glands and reproductive organs is highly debated. For a long time, it has been generally accepted that the parasympathetic innervation to the kidneys was from the vagus nerve. However, some studies proved that there is no convincing neuroanatomical evidence for parasympathetic innervation of the kidney (Jobling, 2011; Kopp, 2011). Retrograde tracing with True Blue and Fast Blue injected into the kidneys in rats resulted in labelled cells in the dorsal root ganglia (T8-L2), nodose ganglia of the vagus nerve, as well as throughout the DVMN and NTS (Donovan et al., 1983). However, the presence of the similar number of labelled neurones in these same locations after injections into the tail vein of rats with denervated kidneys indicates that labelling may be non-specific (Donovan et al., 1983). All other attempts to trace parasympathetic projections to the kidney using retrograde tracers showed that there is no vagal innervation of this organ (Jobling, 2011).

The parasympathetic innervation of the spleen is an ongoing debate. Neuroanatomical and neurochemical evidence in rats demonstrates that neural innervation of the spleen is entirely sympathetic in origin, mainly via the sympathetic splanchnic nerves, and indicates that there is no evidence for parasympathetic or sensory input to the spleen (Nance and Sanders, 2007). Retrograde (Horse radish peroxidase (HRP) and FluoroGold) (Nance and Burns, 1989), as well as transneuronal tracers (pseudorabies virus) (Cano et al., 2001) were used without providing evidence of vagal innervation. Absence of choline acetyltransferase (ChAT) in the spleen, which is a specific marker of cholinergic nerve fibres, further proves absence of parasympathetic innervation (Bellinger et al., 1993). In contrast, studies indicate that the spleen is required for the cytokine regulatory effect of VNS despite all the evidence that the vagus nerve does not directly innervate this organ (Bellinger et al., 1993; Berthoud and Powley, 1993; Koopman et al., 2016; Rosas-Ballina et al., 2008). This may differ between species (Kooijman et al., 2015), however, it has since been revealed that there is indeed no vagal innervation to the spleen and the anti-inflammatory effect of VNS is due to the vagal efferent fibres activating the sympathetic and adrenergic neurones supplying the spleen. This is mediated via activation of alpha-7 nicotinic acetylcholine receptors on sympathetic fibres running in the splenic nerve and affecting cytokine production in the spleen (Browning et al., 2017; Gautron et al., 2013).

Similarly to the spleen, the adrenal glands are innervated sympathetically. Sympathetic preganglionic nerve fibres from neurones in the T10–L1 intermediolateral cell column pass through the sympathetic chain, travel in splanchnic nerves, and directly innervate adrenal medullary chromaffin cells (Câmara and Griessenauer, 2015; Ellis, 2006; Felten et al., 2016). However, some studies indicate that the vagus nerve does supply innervation to the adrenal glands, at least in some species (Berthoud and Powley, 1993; Coupland et al., 1989; Niijima, 1992; Parker et al., 1993). This confusion may be attributed to the role the vagus nerve plays in anti-inflammatory responses as with the spleen, mentioned above (Nance and Sanders, 2007).

There is a debate whether the reproductive organs receive parasympathetic innervation derived from the sacral S2-S4 nerves from the intermediate grey matter of the spinal cord (which also innervate the bladder and lower urinary tract (Chancellor and Yoshimura, 2002)) or from the vagus nerve. The course of the vagus nerve beyond the stomach is very diffuse, thus making it difficult to follow the vagal innervation beyond this point (Câmara and Griessenauer, 2015; Ellis, 2006). There are some studies, however, showing that the vagus nerve may serve as a connection between the reproductive organs and CNS (Collins et al., 1999; Gerendai, 2004; Linares et al., 2013; Pastelín et al., 2017). The argument has been made that the parasympathetic origin to the reproductive organs is from the vagus nerve as opposed to the splanchnic nerves due to the embryonic origin of the ovaries (Brauer and Smith, 2015; Gerendai et al., 2002).

Regarding the afferent function of the vagus nerve, there are somatic and visceral components, as well as a special sensory component (Rea, 2014). Somatic refers to sensation from the skin and muscles. This component is provided by the auricular branch of the vagus nerve which innervates the skin of the back of the ear and external auditory meatus, parts of the external surface of the tympanic membrane, and the pharynx (Rea, 2014). Special sensory component refers to a minor role of the vagus nerve in taste sensation via afferent fibres from the root of the tongue and epiglottis (Câmara and Griessenauer, 2015; Rea, 2014). The visceral sensory component is responsible for the transmission of information from the visceral organs and represents the most interest for the therapeutic applications of vagal stimulation (Blount, 2015; De Ferrari and Schwartz, 2011; Rea, 2014).

### 3.2 Fibre composition and molecular characterisation of vagus nerve

The cell bodies of the majority of vagal afferent neurones reside in the nodose ganglion. Nearly all vagal sensory neurones innervating subdiaphragmatic structures are unmyelinated C-fibres (Prechtl and Powley, 1990). Vagal afferent fibres innervating thoracic structures, such as the respiratory tract and oesophagus, however, are more diverse and comprise about 50% unmyelinated C-fibres and 50% myelinated A-fibres. Vagal sensory A-fibres comprise primarily low-threshold stretch-sensitive receptors. These are referred to as slowly adapting and rapidly adapting receptors in the lungs (Sant’Ambrogio and Widdicombe, 2001) and as tension (mechano)receptors (structurally intraganglionic laminar nerve endings, IGLEs) in the oesophagus (Zagorodnyuk et al., 2003). In addition, a touch- and acid-sensitive A*δ* cough receptor has been described in the trachea (Mazzone et al., 2009). A subset of vagus sensory afferents, including unmyelinated C-fibres and myelinated Aδ-fibres, express TRPV1 (transient receptor potential cation channel subfamily V member 1) – a ligand-gated non-selective calcium permeable ion channel, also known as the capsaicin receptor and the vanilloid receptor 1 (Caterina et al., 1997; Nakagawa and Hiura, 2006). It integrates multiple physical and chemical stimuli, including vanilloid compounds, low pH, and noxious heat. With respect to C-fibres, two distinct phenotypes of capsaicin-sensitive nociceptive C-fibres, referred to as neural crest C-fibres and placodal C-fibres, have been identified in both the respiratory tract and oesophagus (Kollarik et al., 2010; Undem et al., 2004). ATP-sensitive channel P2 × 3 is predominantly expressed by myelinated A-fibres but is also found on a subset of unmyelinated vagal afferent fibres (Hermes et al., 2016). The high conductance calcium-activated potassium channel K(Ca)1.1 was shown to be exclusively expressed by unmyelinated visceral afferent fibres (Li et al., 2011).

In conclusion, there is convincing evidence that the cervical vagus nerve innervates the following organs: the heart, tracheobronchial tree and lungs, oesophagus, stomach, liver, pancreas, small intestine and proximal colon. This includes both parasympathetic efferent innervation, via pre- and postganglionic nerve fibres that synapse within intramural ganglia, and afferent sensory innervation with continuous fibres from brainstem to target organ. This suggests that, at least in principle, it should be possible to label the fascicular path to or from these organs using the available neuronal tracers.

## 4 Neuronal tracing

Neuronal tracers label axon paths to allow delineation of the connectivity in the nervous system (Carter and Shieh, 2015). These are chemical probes that can be classified by the direction they travel within the neurone. Anterograde tracing refers to transport from the cell body to the synapse; retrograde tracing refers to labelling from the synaptic terminal back to the cell body (the opposite direction) (Carter and Shieh, 2015). Some tracers are capable of working in both directions whereas some are restricted to only anterograde or retrograde transport. A revolution in neurobiology in the 1970s was experienced when HRP was introduced as a retrograde tracer for mapping of long-distance neuronal projections (Kristensson and Olsson, 1971; Lavail and Lavail, 1972). When injected into target organ/tissue, HRP is taken up by peripheral autonomic nerves by means of passive endocytosis (Broadwell et al., 1984, 1980). Retrograde transport occurs in small vesicles that tend to fuse and accumulate HRP at high densities in the nerve facilitating the visibility of the final reaction product (van der Want et al., 1997). Visualisation of the transported HRP is achieved during immunocytochemistry; HRP oxidises a substrate in the presence of hydrogen peroxide into an insoluble polymer which is then detectable by light and electron microscopy (Ribatti, 2017). Until then, tracing neuronal pathways often required laborious dissections by hand, or involved Wallerian degeneration – histological description of degenerating axons following lesions of the nerve or brain regions (Waller and Owen, 1850). This lesion-based method was greatly improved by the discovery that degenerating myelinated fibres can be visualised with silver staining technique (Marchi, 1886; Strich, 1968) which gave a driving force to several decades of classical neuroanatomy.

Since the introduction of HRP as a neuroanatomical tracer, many other substances have been identified and used, such as bacterial toxins, plant lectins, viruses and fluorescent molecules (Heimer and Záborszky, 1989; Rajakumar et al., 1993). These tracers may be fluorescent or produce a colorimetric product subsequent to reaction with a substrate. Examples of classical tracers include HRP, biotinylated dextran amines, FluoroGold, Fast Blue and Diamidino Yellow. Modern molecular techniques have enabled the construction of recombinant forms of viruses, such as the adeno-associated virus (AAV), that can be designed to deliver genetically encoded fluorescent protein (for example, green fluorescent protein, GFP) to the neurones for anterograde tracing of long axonal pathways (Chamberlin et al., 1998). The GFP fills the soma and then diffuses down the length of the axon to show targets of the nerve fibres (Carter and Shieh, 2015).

The use of anterograde or retrograde tracing requires the application of a tracer into the tissue of interest in a live animal during a recovery experiment via one of three methods: pressure injection, iontophoretic injection, or mechanical insertion of dye crystals (Köbbert et al., 2000). After an optimal incubation period, the tissue of interest is excised, processed and examined for the presence of the tracer. Neuronal tracing is often combined with other techniques such as immunohistochemistry or electrophysiology (Carter and Shieh, 2015; Köbbert et al., 2000). Classical tracing techniques result in the illumination of direct connections within the nervous system (i.e. from cell body to synapse, or vice versa). However, occasionally, scientists want to study the neural circuitry across multiple synapses. For this, the use of transsynaptic tracers is required. These include radioactively labelled amino acids such as ^3^H-leucine, plant lectins such as wheat germ agglutinin, and viruses such as pseudorabies and herpes simplex viruses (Beier et al., 2011; Carter and Shieh, 2015; Kuypers and Ugolini, 1990; Song et al., 2005; Tuncdemir and Fishell, 2011; Ugolini, 2010). Additionally, proteins such as tetanus toxin can be used for transsynaptic labelling (Cabot et al., 1991; Evinger and Erichsen, 1986; Horn and Büttner-Ennever, 1990; Schwab et al., 1979). However, available methods for transsynaptic labelling have proven to either not be highly efficient (Gerfen and Sawchenko, 1984; Kissa et al., 2002; Schwab et al., 1979) or to have high toxicity (Ekstrand et al., 2008; Lo and Anderson, 2011; Wickersham et al., 2007).

Three uptake mechanisms of tracers exist: active uptake, passive incorporation and intracellular injection (Köbbert et al., 2000). Tracers can enter axons by active uptake via either nerve terminals, allowing for intramuscular injections, or injured neuritic profiles (Köbbert et al., 2000). Both pathways can be successful (Liang et al., 2015); however, some tracers are more readily taken up by one or the other. For example, dextran amines are more efficiently taken up by injured nerves (Choi et al., 2002; Fritzsch and Sonntag, 1991), while Fast Blue is more readily taken up by intact nerve terminals (Hayashi et al., 2007). Substances can also passively diffuse into neurones due to the local concentration gradient (Köbbert et al., 2000). Lastly, if single cells are accessible, tracers can be directly delivered to the cytosol of the neurone. Similar to uptake, transport and distribution of the tracers within the cells take place via active transport in the vesicles or lateral diffusion within the plane of the membrane (Köbbert et al., 2000). Active transport, which appears to be the only efficient mode for longer distances *in vivo*, occurs by means of pH-trapping or ingestion of labelled membrane fragments, followed by tracer accumulation in vesicles and active transport along the cytoskeleton of axons (Abbott et al., 2013; Köbbert et al., 2000). The lateral diffusion results in much smoother labelling of the complete extension of the neural processes and, as it is based on passive diffusion, this can be exploited for the use in a fixed tissue (Godement et al., 1987).

The choice of non-viral neuronal tracers depends on several factors including their labelling efficiency and uptake mechanisms. Several molecules such as carbo-cyanine dyes, beads, dextrans, lectins, amino acids, inorganic fluorescent molecules and bacterial toxins have been previously used as non-viral neuronal tracers. Among these several options, inorganic fluorescent molecules have the key features of an ideal neuronal tracer, which include selectivity, efficiency and availability for multi-colour labelling. Important factors to be considered when choosing a neuronal tracer are: a) duration of time to label the target after the dye application; b) duration of fluorescent labelling after uptake (how long the fluorescent labelling lasts); and c) labelling efficiency of the candidate neuronal tracer which is determined by proportion of cells labelled and intensity of labelling. The time required for the dye to label nerves and neurones innervating a certain tissue/organ varies significantly and depends on the type of the chosen neuronal tracer and structure of the targeted organ undergoing the dye injections. In addition, other factors should be considered. Animal species and the length of neuronal projection from the target organ (e.g., heart vs colon) to the neuronal tracer’s final destination (e.g., nodose ganglia) are the additional critical factors influencing the time of the labelling period. The time sufficient for a neuronal tracer to travel along the nerves and provide consistent labelling outcome needs to be optimised for each particular tracer/target organ/species. For example, Fast Blue is reported to produce labelling as early as two days after injections into mouse airways (Kaelberer and Jordt, 2016) but takes 1–2 weeks to label vagal afferent/efferent projections to abdominal organs in pigs (Zalecki, 2015). An ideal tracer for neuroanatomical mapping would allow labelling the neurones without affecting their function, have a long half-life and specificity. Viral vectors are broadly used for mapping and selective manipulation of neuroanatomical projections within the central nervous system.

In the peripheral nervous system, studies of viral gene transfer have mainly focused on the dorsal root ganglion neurones and studies relating to pain (Glatzel et al., 2000; Mason et al., 2010; Storek et al., 2008; Xu et al., 2003). There are very few studies that report the use of recombinant viral technology in tracing of vagal afferent pathways. Wild-type neurotropic viruses, such as rabies virus from Rabdoviridae and pseudorabies virus, and alpha-herpes virus, are the two main classes of viral transneuronal tracers. Despite good tracing qualities, these viral vectors have high toxicity which typically leads to low levels of expression and variable results. In the last 20 years, there has been a methodological revolution in neuronal tracing with the development of genetically modified viruses and use of other viral species, including AAVs. There is a large number of naturally occurring serotypes of AAVs which differ by capsid proteins and sequences in their inverted terminal repeats – sequences required for replication of viral genome. The different serotypes demonstrate distinct cellular tropism, transduction efficiency, speed of onset of viral gene expression and distribution patterns in infected cells (Aschauer et al., 2013; Haenraets et al., 2017). Use of AAVs for tracing in peripheral nervous system represents a higher level of complexity compared to tracing in CNS. Firstly, it is the dilution factor – when injected into CNS structures, the number of viral particles getting in direct contact with targeted neurones is significantly higher than when the vector is injected into peripheral organ/tissue (Aschauer et al., 2013). Secondly, the majority of AAV serotypes utilise anterograde axonal transport (from neuronal cell body to axonal projections) more efficiently than retrograde (from neural terminals to cell body – the direction which needs to be targeted when tracing organ-specific projections of the vagus nerve) (Aschauer et al., 2013). Recently, new AAV capsids were developed for efficient and non-invasive gene transfer to central and peripheral nervous system; namely, AAV-PHP.eB and AAV-PHP.S (Challis et al., 2019). However, such studies have only been performed in transgenic mice until now. For example, in their work on identification of peripheral neural circuits regulating heart rate, Rajendran et al. (Rajendran et al., 2019) administered Cre-dependent AAV-PHP.S vectors expressing a fluorescent reporter gene into ChAT-IRES Cre mice. Such two-component expression system allows cell type-specific labelling of parasympathetic cholinergic neurones. When injected systemically, the AAV-PHP.S serotype does not allow organ-specific tracing, while injection into organ or tissue does not produce satisfactory results because this serotype is not transported retrogradely. The only AAV variant that permits efficient retrograde access to projection neurones with efficiency comparable to classical retrograde tracers is a newly evolved rAAV-retro (Tervo et al., 2016). This rAAV-retro gene delivery system is now being used on its own or in conjunction with Cre recombinase driver lines to achieve transgene expression for tracing and selective manipulation of neuronal populations projecting to peripheral organs/tissues. A combinatorial viral approach to target and manipulate vagal sensory neurones of the upper gut with the use of rAAV-retro was employed by Han et al. (Han et al., 2018) – the Cre-inducible viral optogenetic construct was injected into the nodose ganglia of mice that received organ-specific peripheral injections of rAAV-retro to express Cre-recombinase. Cre-recombinase was readily detected in enteric neurones of the upper GIT and double-infected neurones were optogenetically manipulated to interrogate sensory pathways involved in gut-mediated reward (Han et al., 2018). This work demonstrates the feasibility of the approach with the use of rAAV-retro in tracing of organ-specific vagal afferent projections. Whether this AAV serotype can be used as successfully for retrograde tracing in large animals as in small rodents, needs to be investigated.

### 4.1 Approaches to non-selectively label all vagal afferents or efferents

To label all abdominal fibres in a rat non-selectively, FluoroGold can be injected via intraperitoneally. After five days, all abdominal vagal afferents are labelled (Zheng et al., 2005). True Blue can be infused intraperitoneally (3 mg per rat) to non-specifically label all vagal afferent and preganglionic efferent neurones innervating viscera (Sterner et al., 1985). Alternatively, red fluorescent dye Dextran–Texas Red can be injected into the nodose ganglion. After 14 days, all sensory (afferent) fibres are labelled with red fluorescent marker (Walter et al., 2009). To label vagal abdominal efferent fibres non-selectively, red fluorescent dye Dextran–Texas Red can be pressure injected into DVMN. Nineteen days later, all subdiaphragmatic efferent fibres are labelled (Walter et al., 2009).

### 4.2 Neuronal tracing of organ-specific projections of vagus nerve

#### 4.2.1 Heart

In large animals, to maximise the efficiency of labelling, the injection points for the neuronal tracers for the heart should be in close proximity or directly into cardiac plexus which is found at the base of the heart. The cardiac plexus is generally divided into superficial and deep portions. The superficial portion lies beneath the aortic arch, just in front of the right pulmonary artery, and is formed by the superior branches of the left sympathetic nerves and the lower superior cervical branches of the left vagus nerve. The deep cardiac plexus lies in front of the tracheal bifurcation behind the aortic arch and is formed by cardiac nerves arising from the cervical ganglia of the sympathetic trunk and cardiac branches of the vagus and recurrent laryngeal nerves. From an electrophysiological perspective, the right-sided nerves tend to supply the sinoatrial (SA) node while the left-sided nerves tend to supply the atrioventricular node (Kapa et al., 2016). In rats, direct injections of HRP (3–5 μl bolus of 30% HRP in saline) into the upper right atrium near SA node or in the mid-ventricular region were performed to trace vagal projections to the heart (Stuesse, 1982). After 24–48 hours of recovery, the labelled cell bodies of vagal preganglionic neurones were predominantly found in the nucleus ambiguus forming a column of cells within the nucleus extending 240 μm in the rostral-caudal direction. The success of this approach suggests it is possible to achieve retrograde labelling of vagal efferents innervating SA node via injections of the tracer into cardiac muscle in close proximity to the SA node. Whether it is feasible in large animals, still needs to be tested.

#### 4.2.2 Trachea

Vagal cough receptor sensory neurones in the trachea can be visualised by FluoroGold labelling: in guinea pigs, 10 μl of 4% FluoroGold was injected into the rostral extrathoracic trachea lumen on to the mucosal surface. Seven days post-injection, about 3% of neurones within the nodose ganglion were labelled with FluoroGold (Mazzone and McGovern, 2008).

#### 4.2.3 Oesophagus

Vagal afferent neurones were successfully traced using AAV2/8 viral vectors in the guinea pig (Kollarik et al., 2010). The eGFP encoded by adeno-associated virus vectors was transported to both the central and peripheral terminals when the virus was injected into the nodose ganglion. AAV2/8 vectors were also efficiently taken up by vagal nerve terminals in the visceral tissue (when injected into oesophageal wall) and retrogradely transported to the cell bodies (residing in nodose ganglion). A total of 20 μl of AAV2/8 vector (10^12^ viral particles/ml) were injected directly into the cervical oesophageal wall. Seven weeks after injection, GFP was expressed in right and left nodose ganglia (Kollarik et al., 2010). In rats, Fast Blue was used for retrograde labelling of vagal afferents in subdiaphragmatic oesophagus. Four injections each containing 2 μl of 4% Fast Blue solution were applied into the oesophageal muscle below the diaphragm (Banerjee et al., 2009). Two weeks after injection, the Fast Blue-labelled cell bodies were found in nodose ganglion (Banerjee et al., 2009).

#### 4.2.4 Pancreas

Vagal afferent projections to the pancreas mostly innervate the head of the pancreas and to the lesser extent the tail (Fasanella et al., 2008), and the number of neurones projecting from the left nodose ganglion is almost twice as high as from the right (Fasanella et al., 2008). Pancreatic sensory afferents can be labelled by direct injection of the tracer into the parenchyma of the organ. In mice, 5 μl of 2% Cholera toxin subunit B (CTB) conjugated to Alexa Fluor 488/555/647 were injected across three sites into the duodenal lobe pancreatic parenchyma using glass micropipette, and four days after the injection the tracer was visualised in the nodose ganglion (Fasanella et al., 2008). Alternatively, tracing can be performed with True Blue: 5–10 μl of True Blue in distilled water (5%) was injected into the duodenal and splenic lobes of the pancreas at 8–12 loci, and seven days after injection the labelled cell bodies of pancreatic sensory afferents were visualised in nodose ganglion (Su et al., 1987).

#### 4.2.5 Liver

About 30% of the fibres in the common hepatic branch are of non-vagal (adventitial) origin (Prechtl and Powley, 1990). Sensory vagal fibres in the liver are restricted to the stroma surrounding triads of hepatic vasculature and bile ducts, and to extrahepatic portions of the portal vein and bile ducts. For vagal afferent innervation, retrograde and anterograde tracing studies in the rat have clearly shown that only a minor portion of the common hepatic branch innervates the liver area, while the major portion descends in the gastroduodenal branch toward the duodenum, pancreas, and pylorus. Hepatic paraganglia, bile ducts, and the portal vein receive the densest vagal afferent innervation (Berthoud, 2004). Studies with retrograde tracer HRP injections (40 μl of 21% HRP) into various compartments of liver revealed that the smallest number of labelled vagal afferent neurones is found following direct injections into hepatic parenchyma. Injection into the hilus area was more effective, while injections into common bile duct was 10-fold more efficient (Magni and Carobi, 1983). These findings led to the conclusion that the majority of axons travelling in the hepatic branch terminate in bile ducts. More than half of the fibres travelling in hepatic branch innervate the pylorus and first part of duodenum (Berthoud, 2004).

#### 4.2.6 Gastrointestinal tract: small and large intestine

Majority of the sensory neurones that innervate intestinal villi (mechano- and chemoreceptors) contain the receptor GPR65 (Williams et al., 2016). Selective activation of these neurones causes a powerful blockade of gastric contractions without impacting breathing or heart rate, which are also under vagal control. In a fixed tissue from murine embryos and neonates, DiI was used to label and trace the vagus nerve supplying the GIT; DiI crystals were inserted directly into the anterior trunk of subdiaphragmatic vagus nerve (Murphy and Fox, 2007). After allowing three weeks for diffusion of the Dil, the GIT was imaged for the presence of the tracer that anterogradely labelled vagal afferents.

#### 4.2.7 Stomach and duodenum

In rats, vagal innervation of stomach and duodenum can be traced with True Blue or CTB-conjugates. 5–10 μl of True Blue in distilled water (5%) was injected into anterior and posterior walls of the stomach at 8–12 loci with a Hamilton microsyringe. Histology performed seven days after the dye injection revealed labelled neuronal cell bodies in the nodose ganglia (Su et al., 1987). Fluorescent conjugate of CTB with Alexa Fluor 555 was injected into the wall of the stomach and duodenum using glass capillary loaded with 10 μl of tracer (0.5% solution of CTB-555 in saline). One week later, the labelled cell bodies of afferent neurones can be quantified in the nodose ganglia (Diepenbroek et al., 2017).

#### 4.2.8 Mucosal tracing in the stomach

The vagal sensory afferents innervating stomach mucosa in rats can be labelled with CTB-Alexa Fluor conjugates. Animals have to be fasted to minimize the amount of food in the stomach, and mucolytic agent has to be applied prior to the neuronal tracer to maximise the tracer uptake. 0.5% CTB-555 was injected into the lumen of the stomach (60 μl) using a glass capillary. After one week, the labelled cell bodies of sensory neurones were visualised in the nodose ganglia (Diepenbroek et al., 2017).

#### 4.2.9 Colon

As for the stomach and duodenum, vagal innervation of the colon in rats can be traced with CTB-555. A 0.5% solution of CTB-555 was injected into ventral and dorsal sides of the first 2 cm of ascending colon (distributed over 10 injection sites, 5 μl of the tracer in total) and the labelled cell bodies of afferent neurones were visualised in the nodose ganglion (Diepenbroek et al., 2017).

Regarding neuronal tracing in large animals, the majority of studies of vagal sensory innervation in pigs is performed with the use of retrograde fluorescent tracer Fast Blue: 20 μl of 5% aqueous suspension of Fast Blue was injected with a Hamilton microsyringe across 4–5 sites into the organ/tissue of interest in young pigs (body weight approx. 20 kg), and histology was performed 10–15 days after injection (Zalecki, 2015). The techniques suitable for neuronal tracing in small laboratory animals often fail in the larger species. For example, no sensory parasympathetic neurones in nodose ganglion were labelled when using CTB-HRP in pigs (Zalecki, 2015).

## 5 Conclusion

The vagus nerve innervates numerous organs including the heart, tracheobronchial tree and lungs, oesophagus, pancreas, liver, stomach, small intestine and proximal colon, as well as the muscles associated with the pharynx and larynx. The vagal innervation to the spleen, kidneys, adrenal glands and reproductive organs is highly debated. Sensory fibres of the vagus nerve run uninterrupted, with no synapsing, directly from the target effectors to the solitary and spinal trigeminal nuclei of the brainstem where the afferent information is processed and relayed to the DVMN and the nucleus ambiguus. Preganglionic neurones residing in these nuclei project to the terminal intramural ganglia within or near the target organs where they synapse on the postganglionic neurones that project from the intramural ganglia to the target effector, or to the specific target tissue within the organ.

Neuronal tracing has been successfully implemented to trace vagal innervation of all the thoracic and abdominal organs. The majority of the studies were performed in small laboratory animals for tracing of the afferent neuronal projections from the innervated organ to the cell bodies residing in nodose ganglia. For neuronal tracers utilizing active mechanisms of axonal transport, this results in labelling of the cell bodies exclusively. For efficient axonal labelling along the entire nerve, the passively diffusing tracers or viral vectors need to be used. Even with the use of non-transsynaptic neuronal tracers, tracing of the fascicular anatomy of the afferent fibres to determine the arrangement and localisation of fascicles within the cervical vagus nerve appears to be feasible. These fibres can be retrogradely traced when the tracer is injected into parenchyma of the innervated organ or into the smooth muscle wall or submucosal layer of the organ of GIT (stomach, duodenum, ileum, and jejunum). When tracing efferent fibres, the tracer has to be injected into the brainstem nuclei where the cell bodies of vagal preganglionic neurones reside (DVMN or nucleus ambiguus) taking into account the viscerotopic organisation of the brainstem nuclei. However, the organ specificity of such central approach to label vagal efferent fibres is unlikely to reach 100% due to significant overlap of viscerotopic columns of vagal preganglionic neurones within brainstem nuclei (Berthoud et al., 1991). Tracing of efferent fibres using monosynaptic tracers will result in the labelling termination in the intramural ganglia. Otherwise, transsynaptic tracers, although not optimal, should be used.

A good starting point for using neuronal tracers to map the fascicular anatomy of nerves would be to begin with those used successfully in the literature (Table 1). However, it would be advantageous if the same neuronal tracer would work for each organ of interest, which requires method development and optimisation.

**Table 1:** Neuronal tracers used in literature to trace vagal projections to various organs.

Organ/effector	Neuronal tracers used in literature	Reference
Heart	HRP	Stuesse, 1982
	AAV-PHP.S	Rajendran et al., 2019
Tracheobronchial tree and lungs	FluoroGold	Mazzone and McGovern, 2008
Oesophagus	Fast Blue	Banerjee et al., 2009
	AAV2/8	Kollarik et al., 2010
Pancreas	True Blue	Su et al., 1987
	CTB conjugates	Fasanella et al., 2008
Liver	HRP	Magni and Carobi, 1983
GIT	DiI	Murphy and Fox, 2007
	rAAV-retro	Han et al., 2018
Stomach and duodenum	True Blue	Su et al., 1987
	Fast Blue	Zalecki, 2015
	CTB conjugates	Diepenbroek et al., 2017
Colon	CTB conjugates	Diepenbroek et al., 2017

Neuroanatomical tracing will allow mapping of the fascicular anatomy of the vagus nerve from the numerous target effectors to the cervical level. However, additional information on the functional anatomy and potential reflex activity of the vagus nerve will also need to be taken into account. Such a map of the functional anatomical organisation of the vagus nerve will enable selective stimulation of fascicles innervating the organ/tissue of interest, which will ultimately help to avoid the off-target effects so frequently experienced during VNS and improve its therapeutic efficacy.
